# Promoción de la salud nutricional en un escenario educativo de una región vulnerable del Perú

**DOI:** 10.1016/j.aprim.2023.102676

**Published:** 2023-05-30

**Authors:** Mercedes Acosta-Román, Liszeth Paola Cerna-Ruiz, Charles Frank Saldaña-Chafloque, Miguel Ángel Yglesias-Jauregui

**Affiliations:** Universidad Nacional Autónoma de Tayacaja Daniel Hernández Morillo, Pampas, Tayacaja, Huancavelica, Perú

Es importante reconocer que la educación para la salud, según la Organización Mundial de la Salud, es la estrategia principal que orienta y organiza las actividades en la temática de salud para conseguir los cambios de conducta en la población, influyendo en acertados conocimientos en salud y prácticas saludables en búsqueda de su bienestar^1^. Uno de los principales temas que aborda el personal de salud en el escenario educativo es la alimentación saludable, siendo esencial que los niños y adolescentes posean conocimientos preventivos y así resulte en ellos un cambio positivo a su crecimiento y desarrollo^2^. Según últimos reportes, México enfrenta el gran problema de salud en los niños de 6 a 11 años con el sobrepeso u obesidad, de cada 10 escolares 3 lo presentan, esto según sus encuestas nacionales de salud y nutrición^3^. Siendo también en Perú un problema sanitario en los adolescentes, resultando que en un estudio con una muestra de 242 adolescentes de 3 regiones del Perú se encontró que los varones presentaron sobrepeso y obesidad en un 25,7% y las mujeres en un 12,1%^4^. Por todo lo mencionado anteriormente, esto dio inicio a realizar un estudio en una institución educativa estatal de nivel secundario de una de las regiones más vulnerables del Perú, que es Huancavelica; el objetivo de este estudio fue identificar el efecto de la promoción de salud en alimentación y nutrición en los alumnos de la Institución Educativa Túpac Amaru II del anexo de Pampa Blanca del Distrito de Daniel Hernández 2022; fue de tipo cualitativa, de diseño demostrativo y actividades grupales sobre educación en salud en el contexto de reducción del COVID-19 durante el 2022. El estudio fue en el Distrito de Daniel Hernández, que pertenece a la provincia de Tayacaja, del departamento de Huancavelica, el año 2022. Teniendo en cuenta la cantidad de alumnos del nivel secundario, y sea representativa la muestra fue en su totalidad la población de 74 estudiantes, repartidos en 5 grados; las edades fluctúan entre 11 a 16 años, con un promedio de edad de 13,5. Respecto al sexo, mayor predominancia en el femenino, con un 58% y el masculino con un 42%; se realizaron 3 capacitaciones presenciales: beneficios de una buena alimentación, riesgo del consumo de productos ultraprocesados y los 5 grupos de alimentos principales que conforman una ingesta adecuada. Durante las capacitaciones se respetaron el distanciamiento, las medidas de bioseguridad y el aforo respectivo; se usó material digitalizado en las capacitaciones del primer y segundo tema, y material escrito para el tercer tema, siendo una sesión demostrativa; para reforzar el conocimiento brindado al final se realizó una retro alimentación y el seguimiento mediante llamadas. Los resultados finales fueron excelentes; se usó la escala vigesimal para los puntajes de las notas, con los rangos de muy malo en calificaciones de 0 al 10, malo de 11 al 13, regular de 14 al 16, bueno de 17 al 19 y excelente 20 de nota; los resultados del pretest fueron muy malo un 27%, malo un 27%, regular un 26% y bueno con un 20%, mejorándose las calificaciones del postest, dando regular un 18%, bueno un 58% y excelente un 24%. Respecto a la encuesta de satisfacción que fue aplicada en los participantes para calificar las capacitaciones, arrojó que fueron buenas en un 78% y excelente en un 22%. Concluyendo que las escuelas son los mejores escenarios para la implementación de intervenciones educativas en salud, porque los mismos alumnos adquieren los conocimientos a un corto plazo y el cambio de conducta saludable a un mediano y largo plazo, lo cual es el objetivo de todo personal salubrista.

## Consideraciones éticas

Se tiene el consentimiento informado de los individuos participantes del estudio.

## Financiación

Autofinanciado.

## Conflicto de intereses

Se da a conocer que no existe conflicto de interés.

## References

[1] Hernández J., Jaramillo L., Villegas J., Álvarez L., Roldan M., Ruiz C. (2020). La educación en salud como una importante estrategia de promoción y prevención. Arch Med..

[2] Gonzales V., Greca I. (2022). Estrategias de prevención desde la escuela: implementación de una propuesta de enseñanza sobre nutrición humana basada en metodologías activas de aprendizaje. Perspect en Nutr Humana..

[3] Almeida-perales C., Solano-hernández B.I. (2022). Educación para un entorno alimentario escolar saludable. El caso de una primaria en Zacatecas, México. Hacia la Promoción la Salud..

[4] Rivas S., Saintila J., Rodríguez M., Calizaya Y., Javier D. (2021). Conocimientos, actitudes y prácticas de alimentación saludable e índice de masa corporal en adolescentes peruanos: un estudio transversal. Rev Española Nutr Humana y Dietética..

